# Skeletal stem cells: origins, definitions, and functions in bone development and disease

**DOI:** 10.1093/lifemedi/lnac048

**Published:** 2022-12-08

**Authors:** Heng Feng, Bo Jiang, Wenhui Xing, Jun Sun, Matthew B Greenblatt, Weiguo Zou

**Affiliations:** State Key Laboratory of Cell Biology, Shanghai Institute of Biochemistry and Cell Biology, CAS Center for Excellence in Molecular Cell Science, Chinese Academy of Sciences, University of Chinese Academy of Sciences, Shanghai 200031, China; State Key Laboratory of Cell Biology, Shanghai Institute of Biochemistry and Cell Biology, CAS Center for Excellence in Molecular Cell Science, Chinese Academy of Sciences, University of Chinese Academy of Sciences, Shanghai 200031, China; State Key Laboratory of Cell Biology, Shanghai Institute of Biochemistry and Cell Biology, CAS Center for Excellence in Molecular Cell Science, Chinese Academy of Sciences, University of Chinese Academy of Sciences, Shanghai 200031, China; Department of Pathology and Laboratory Medicine, Weill Cornell Medicine, New York, NY 10065, USA; Department of Pathology and Laboratory Medicine, Weill Cornell Medicine, New York, NY 10065, USA; Research Division, Hospital for Special Surgery, New York, NY 10065, USA; State Key Laboratory of Cell Biology, Shanghai Institute of Biochemistry and Cell Biology, CAS Center for Excellence in Molecular Cell Science, Chinese Academy of Sciences, University of Chinese Academy of Sciences, Shanghai 200031, China; Institute of Microsurgery on Extremities, Shanghai Jiao Tong University Affiliated Sixth People’s Hospital, Shanghai 200233, China

**Keywords:** bone development and repair, skeletal stem cells (SSCs), the SSC niche, cell fate regulation, osteoblasts

## Abstract

Skeletal stem cells (SSCs) are tissue-specific stem cells that can self-renew and sit at the apex of their differentiation hierarchy, giving rise to mature skeletal cell types required for bone growth, maintenance, and repair. Dysfunction in SSCs is caused by stress conditions like ageing and inflammation and is emerging as a contributor to skeletal pathology, such as the pathogenesis of fracture nonunion. Recent lineage tracing experiments have shown that SSCs exist in the bone marrow, periosteum, and resting zone of the growth plate. Unraveling their regulatory networks is crucial for understanding skeletal diseases and developing therapeutic strategies. In this review, we systematically introduce the definition, location, stem cell niches, regulatory signaling pathways, and clinical applications of SSCs.

## Introduction

The development, maintenance, and repair of skeleton require the self-renewal and differentiation of SSCs [1]. The identification of SSC markers is the first step to study stem cell function. In recent years, great progress has been made in defining bona fide SSCs through a combination of fluorescence activated cell sorting (FACS) and lineage reporters. SSCs supplant the prior concept of “Mesenchymal stem cells (MSCs).” While MSCs had issues with an inconsistent definition in the literature, they were most stringently considered as cells display clonal multipotency and self-renewal *in vivo* [2, 3]. Colony-forming assays (CFU-Fs) and *in vitro* tri-lineage differentiation assays (the ability to generate osteoblasts, chondrocytes, and adipocytes) were widely used to define MSCs. However, these properties are widely distributed among skeletal cells, due in part to their inherent plasticity. Accordingly, there is a very poor correlation between the *in vitro* presence of these properties and the in *vivo* function of corresponding cellular populations. Additionally, many MSC studies also had issues with highly heterogeneous populations of cells being used as input material [4]. Due to these limitations, the MSC concept should be largely considered as supplanted by the more recently developed SSCs.

A major breakthrough came in the field is the identification of SSCs as bearing the surface markers CD45^−^/TER119^−^/Tie2^−^/Thy^−^/6C3^−^/CD105^−^/AlphaV^+^/CD200^+^, making it possible to isolate SSCs through FACS from several anatomic sites. SSCs are then defined by Chan et al. as capable of self-renewal and giving rise to non-stem progenitor cells, like pre-bone cartilage and stromal progenitor (pre-BCSPs) and BCSPs. Also, they have the ability to generate organoid structures containing bone, cartilage, and stroma when transplanted into the mouse kidney capsule. It is also useful to evaluate the differentiation hierarchy of these populations when defining SSCs. While many cell types can form bone organoids after transplantation, albeit sometimes with differences in the tissue types present in these organoids, there is still a clear differentiation hierarchy among populations under consideration that clarifies which cell types are the most primitive in the lineage. For instance, when isolated from bulk long bone digests, mSSCs, pre-BCSPs, and BCSPs all generate bone, cartilage, and hematopoietic supportive stroma, however while mSSCs can generate CD105^+^ BCSPs after transplantation, BCSPs are unable to generate mSSCs [5]. Notably, while these criteria were used for initial definition of SSCs, there appear to be distinct subsets of SSCs that reside at specific anatomic sites that may require modification of these initial criteria. For instance, CTSK-lineage periosteal stem cells appear to be unable to form adipocytes under basal conditions, in keeping with the physiologic paucity of adipocytes in the periosteum [6]. Thus, not all SSCs will generate all of the mature cell types comprising bone.

When cells are isolated and cultured *in vitro*, their cellular properties and surface marker profiles can be altered by the *in vitro* microenvironment, accounting for the clear discrepancies between *in vivo* and *in vitro* differentiation assays. Therefore, two major lines of experimentation have emerged to study SSCs. In the first, specific cell populations are isolated by FACS, typically with a broad panel of cell surface markers such as the Chan et al. markers above. These cells can then be subjected to transplantation into either a heterotopic site, such as the kidney capsule, mammary fat pad, or intramuscular space, or an orthotopic site, most typically the femur marrow cavity. This is then typically followed by either determination of the types of tissue or bone organoid generated by the input population, demonstrating generation of functional osteoblasts, chondrocytes, and/or adipocytes. Alternatively, this method can be used for differentiation hierarchy studies by re-isolating the transplanted cells and determining which cell types each input cell type is able to generate. Typically, only this method is able to distinguish the distinct functions of specific cell types within a given lineage, as *in vivo* lineage tracing methods, the other major approach, only inform about the overall function or location of the entire lineage and cannot directly distinguish between the contributions of specific cell types within that lineage. Thus, transplantation-based methods are an indispensable part of defining specific cell types in bone, including SSCs.

**Figure 1.**
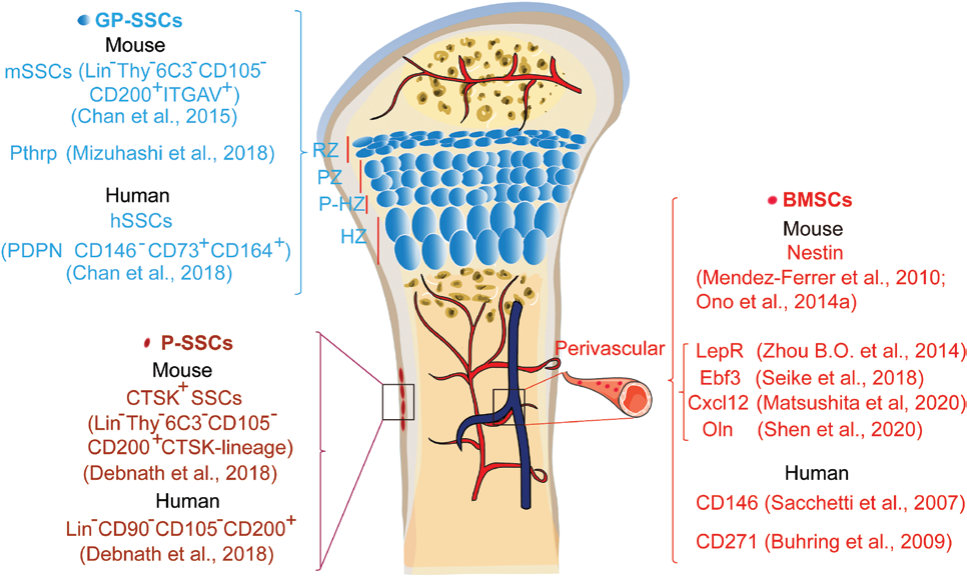
The location and makers of skeletal stem cells (SSCs) and BMSCs. Various subpopulation of SSCs have so far been identified using FACS, Cre/Loxp, or CreER/Loxp system. Bone marrow is rich in blood vessels and many BMSCs located in the bone marrow are perivascular [17–24]. The growth plate include resting zone (RZ), proliferation zone (PZ), pre-hypertrophic zone (P-HZ), and hypertrophic zone (HZ). CD45^−^/TER119^−^/Tie2^−^/Thy^−^/6C3^−^/CD105^−^/AlphaV^+^/CD200^+^ cells from the growth plate of mice are defined as mSSCs while PDPN^+^CD146^−^CD73^+^CD164^+^ cells from growth plate of human are defined as hSSCs [5, 25]. Pthrp^+^ SSCs are located in the RZ of growth plate [26]. Periosteum contains SSCs with high bone regenerative potential [6].

*In vivo* lineage tracing compliments transplantation-based methods by providing insight into the differentiation and function of a given lineage, typically as defined by a single genetic reporter, in an *in vivo* system without surgical manipulation [7–15]. Most commonly, Cre/Loxp-mediated transgenic mice are used for this purpose in combination with a separate reporter allele, where Cre expression under a specific regulatory elements drives the reporter to trigger expression of a GFP or RFP variant in all Cre-expressing cells and their progeny [16] (Fig. 1 and Table 1). However, possibly with the exception of PTHrP-based Cre systems that mark a population of growth plate-resident stem cells, it largely remains unclear whether any given Cre line provides selective labeling of SSCs, with many Cre lines likely labeling several skeletal lineages simultaneously [26]. In this respect, combining FACS together with this genetic reporter approach can be helpful, as additional cell surface markers can assist with resolving the labeled cell types into homogenous populations. This Cre-based *in vivo* lineage can also be used to determine the overall function of the lineage marked by this Cre line, typically by either using a Cre-activated diptheria toxin system to delete this cell type or by deleting a gene functionally required for generating key mature cell types produced by this lineage. For instance, osterix is a key transcription factor for osteoblast differentiation and deletion of it can be used to determine the contribution of the lineage to the osteoblast pool and skeletal mineralization [32].

**Table 1.** Summary of reported cell lineage of skeletal stem/progenitor cell and stromal cells

Reported markers	Location	induction	Chase	Overlapped Markers	Cell lineages contribution	References
Prrx1-Cre	WB	–	E16.5	–	All cells derived from limb bud mesoderm	[7]
	BM	–	Adult	PDGFRα, SCA1, CXCL12, LepR	S, Ob, A, C	[8, 9]
	PO	–	5–8W	PDGFRα, Grem1, CXCL12, LepR, Nestin, NG2	Ob, C, S (callus)	[9, 27]
Prrx1-CreER	GP/BM/PO	TAM P19-23	E26	–	Cells in BM, GP and PO	[10]
		TAM P52-53	P59	–	Cells in BM, GP and PO	[10]
LepR-Cre	BM	–	2, 6, 10, 14M	PDGFRα, PDGFRβ, CD51, CD105, Cxcl12, Scf, Sca1	Ob, C, S Ob, C, S (transplantation)	[19]
	PO	–	2, 8W after frature	–	Ob, C (callus)	
LepR-CreER	BM	TAM P1-3	After 1D	PDGFRα	S (low)	[28]
		TAM P1-3	After 2M		Ob (low), S	
		TAM at 2 M	After 1D		S	
		TAM at 2 M	After 4M		Ob (high), S, A (high) Ob, C (callus)	
Mx1-Cre	BM	pIpC at 6-8W	After 20D or 6M	PDGFRα, CD105	Ob	[11]
	PO	pIpC at 3M	After 2M	Cxcl12, Runx2, α-SMA,	S	[29]
		pIpC at 6M	After 16W	LepR, Grem1, CD200,	S	
		pIpC at 2M	2W post injury	PDGFRα, CD106	Ob, C (callus)	
Cxcl12-CreER	BM	TAM at P21	at P28	LepR, Cxcl12 (high)	S	[22]
		TAM at 8W	at 9W		S	
		TAM at 8W	3M, 6M, 1Y, 1.5Y		S, Ob	
		TAM at 6-10W	2W post injury		S, Ob, C	
Osx-CreER	BM	TAM at E13.5	After 1D	PDGFRα, PDGFRβ, Nestin, LepR	S (Perichondrum)	[12]
		TAM at E13.5	After 2W, 13W		S, Ob	
		TAM at P5	After 1D, 3W, 24W		S, Ob	
		TAM at 8W	After 1D, 2W, 7W		S, Ob	
		TAM at P5	D8 after fracture at 32W		C	
Oln-CreER	BM	TAM at 2M	After 3D, 2W, 1M, 2M	LepR	S, Ob	[20]
		TAM at 2M	After 6M, 12M		S	
		TAM at 2M	2W post injury		Ob	
Grem1-CreER	GP/BM/PO	TAM at P1	at 6W	CD105, Sca1, Nestin, PDGFRα	S,C	[30]
		Adult TAM	After 1W		S, Ob, C	
		Adult TAM	7DPF		S, Ob, C (callus)	
Pthrp-CreER	GP	TAM at P6	at D12, D15, D18, D36	CD105, CD200, CD51	C	[26]
		TAM at P6	at 2M, 3M, 6M, 1Y		S, Ob, C	
Col2a1-CreER	GP	TAM at P3	at P5	CD73	C	[31]
		TAM at P3	at P28		S, Ob, C	
		TAM at P0	at P10		S, Ob, C	
		TAM at P30	at P40		S, Ob, C	
Acan-CreER	GP/BM	TAM P1-3 or at 2M	After 1D		S, C	[28]
		TAM P1-3	After 1M		S, Ob, C	
		TAM at 2M	After 2M		S, Ob, C	
FoxA2-CreER	GP	TAM at P3-4, P7-8, P13-14	3M		Ob, C	[13]
Ctsk-Cre	PO		E14.5, P10	PDGFRα (low), CD146(low), Grem1, Nestin, Runx2, Ocn, Alpl, CD200, CD105	Ob, C, S in the callus	[6]
			D7,15, 32		Ob after transplantation	
α-SMA-CreER	PO	Adult TAM	7DPF	PDGFRα, PDGFRβ, Sca1, CD51, CD90, CD105, CD200	S, Ob, C (callus)	[14]
PDGFRα-CreER	PO	TAM at 2 M	1, 3, 7, 14DPF at 10W	PDGFRβ, Ctsk, Sox2, α-SMA, Myc, Pou5f1, CD44, CD90, Sca1	S, Ob, C (callus)	[15]

Abbreviations: WB: whole bone; BM: bone marrow; PO: periosteum; TAM: tamoxifen; D: day (s); W: week (s); M: month (s); Y: year; E: embryo; P: postnatal; DPF/WPF:days post fracture/weeks post fracture; pIpC: polyinosinic:polycytidylic acid; A: adipocyte; Ob: osteoblast; C: chondrocyte; S: stromal cells

Lineage tracing and single-cell sequencing have widened and deepened the knowledge of multiple types of SSCs, including growth plate skeletal stem cells (GP-SSCs), periosteal skeletal stem cells (P-SSCs), and BMSCs [33, 34]. The origin of SSCs in these three locations remains to be elucidated. In view of these studies, this review aims to collectively present and compare reported SSC populations in mice and humans. We also discuss the role of niche factors in maintaining SSCs and the regulatory signaling network of SSCs in skeletal development and repair to aid the development of targeted cellular therapies for skeletal diseases.

## BMSCs

BMSCs in the bone marrow stroma are heterogeneous, including long-lived progenitor populations. C-X-C motif chemokine 12 (CXCL12)-abundant reticular (CAR) cells are a leading cell population of BMSCs, which are perivascular and secrete CXCL12/SDF1 and stem cell factor to maintain hematopoiesis [35, 36]. Nestin is a marker of neural stem/progenitor cells and has been used to mark a subet BMSCs using the Nestin-GFP transgenic mice. Nestin-GFP labels perivascular, clonal cells in the bone marrow. Interestingly, depletion of Nestin^+^ BMSCs inhibits hematopoiesis [17]. However, Nestin-CreER labels predominantly CD31^+^ endothelial cells in the developing bone marrow induced by tamoxifen, which has given rise to much controversy over whether Nestin is an appropriate SSC marker [18, 19]. The leptin receptor (LepR)-Cre labels adult BMSCs and largely overlap with CAR cells. LepR-Cre-labeled cells overlap with Nes-GFP, but the relationship between them is unclear and likely nestin labels a broader pool of cells aside from LepR^+^ cells based on reporter studies [18]. LepR^+^ cells are mainly concentrated the perivascular area and they are capable of producing bone marrow adipocytes. LepR^+^ cells contributed to the pool of adipocytes in the bone marrow of 2-month-old mice and the contribution increased with age. As LepR^+^ cells only emerge postnatally, they are not involved in the prenatal developmental or early postnatal generation of osteoblasts, but their contribution to the osteoblast pool increases with increasing age [19]. Recent single-cell transcriptome analysis of bone marrow cells revealed that LepR^+^ cells can be subdivided into different subpopulations [37–39]. These findings suggest LepR^+^ cells are heterogeneous, including separate cell types poised for either osteogenic or adipogenic differentiation. Osteolectin (Oln) is a growth factor promoting osteogenesis and distinguishes peri-arteriolar LepR^+^ cells skewed toward osteogenic differentiation from peri-sinusoidal LepR^+^ cells. Peri-arteriolar LepR^+^Oln^+^ cells are osteogenic progenitors that contribute to fracture healing and declines during aging [20]. Ebf3-CreER labeled BMSCs highly overlap with CAR cells can contribute to osteoblasts in physiological and regenerative conditions as well as adipocytes [21].

CAR cells are a heterogeneous pool of cells that highly overlap with both LepR and Ebf3, indicating that at least to an initial approximation all three markers largely delineate the same pool of cells. Accordingly, as with LepR^+^ cells, CAR cells are heterogeneous as they can be separated by two distinct groups of pre-adipocyte-like reticular (adipo-CAR) cells and pre-osteoblast-like (osteo-CAR) cells by single-cell RNA sequencing [39]. A recent study used Cxcl12-CreER mice to trace peri-sinusoidal BMSCs, a cell population that maintains cortical bone homeostasis and participate in osteoblast formation during bone repair [22]. Interestingly, Cxcl12^+^Alpl^+^ Osteo-CAR cells showed a non-sinusoidal localization, which is congruent with the separate findings that peri-arteriolar LepR^+^Oln^+^, but not peri-sinusoidal LepR^+^ cells, are osteogenic.

Additionally, bone morphogenetic protein (BMP) antagonist Gremlin1 (Grem1) labels cells mainly in the metaphysis. Whereas, Grem1-labeled cells are enriched for CFU-Fs and but do not overlap with Nestin, LepR, and Cxcl12, indicating that Grem1 denotes a distinct subset of stromal cells in the marrow [30].

Bone marrow is an important hematopoietic organ and BMSCs support hematopoiesis in addition to directly serving as a source of osteogenic cells. Cxcl12^+^ CAR cells, as well as CAR cell that labeled by LepR-Cre, Prrx1-Cre, Ebf3-CreER, etc., are critical for the maintenance of hematopoietic stem cells (HSCs) [21, 36, 40]. Overall, further studies are needed to analyze the heterogeneity of BMSCs and the function of distinct BMSC subsets with more specific markers and genetic tools.

## Growth plate-derived skeletal stem cells (GP-SSCs)

Bone development has long been known to proceed by two stereotyped sequence of events: intramembranous ossification and endochondral ossification. Long bones develop mainly through endochondral ossification. During the process of endochondral ossification, stem cells originate from the mesoderm condense and differentiate into chondrocytes. The chondrocytes secrete cartilage matrix and then undergo hypertrophy and vascular invasion, which triggers the differentiation of osteoblasts and the eventual formation of the bone marrow cavity. Blood vessels infiltrate into the hypertrophic chondrocytes, which contribute to the development of the primary ossification center. Regions of what later become the growth plate also become distinct at this time within the chondroepiphysis and consist of four structures: the resting zone, the proliferative zone, the pre-hypertrophic zone, and the hypertrophic zone. The primary ossification centers are eventually replaced by the formation of secondary ossification centers (SOC) [41].

In the hypertrophic zone, hypertrophic chondrocytes are terminally differentiated state of growth plate chondrocytes and adjacent to bone and bone marrow [42]. However, a series of studies have shown that chondrocytes in the growth plate can transdifferentiate into osteoblasts [43, 44]. The growth plate contains SSCs labeled by Sox9, Col2a1, or Aggrecan (chondrogenic markers), though these markers appear to also be expressed by SSC populations outside of the growth plate. This process of transdifferentiation of growth plate chondrocytes supplies osteoblasts, adipocytes, and BMSCs by lineage tracing experiments, however it remains unclear whether this is a major pathway for osteoblast generation or whether the contribution of this process is more limited [45].

Cell surface markers of GP-SSCs in mouse (CD45^−^/TER119^−^/Tie2^−^/Thy^−^/6C3^−^/ CD105^−^/AlphaV^+^/CD200^+^) and human (PDPN^+^CD146^−^CD73^+^CD164^+^) are well defined [5, 25, 26]. It remains unclear whether these human and murine sets of markers denote what is fundamentally the same population in both species or a distinct population. GP-SSCs were obtained from the growth plate of the mouse femur, but it is important to note that cells labeled by the same set of markers were also found to be located outside the growth plate. PDPN^+^CD146^−^CD73^+^CD164^+^ human GP-SSCs, which have the ability to self-renew *in vitro* (the ability to continuously generate CFU-Fs), were transplanted into the kidney capsule in mice and were able to generate multilineage bone organoids containing bone, cartilage, and stroma.

Many of the insights into the biology of GP-SSCs have come from the use of Pthrp-based genetic reporters that identify GP-SSCs in the resting zone of the growth plate. Pthrp-CreER^+^ cells are the primary source of growth plate chondrocytes after several months of labeling [26, 31]. Induced death of Pthrp^+^ cells by diphtheria toxin can block bone elongation. Furthermore, Pthrp^+^ cells were shown to be involved in the formation of Col1a1^+^ osteoblasts and Cxcl12^+^ BMSCs, demonstrating *in vivo* that chondrocytes in the resting zone can be converted into hypertrophic chondrocytes and eventually transdifferentiated into osteoblasts and stromal cells. Interestingly, Pthrp-CreER-labeled cells are not transformed into bone marrow adipocytes *in vivo* during homeostasis, however, it cannot ruled out that they can differentiate into adipocytes under stressful condition [26]. Shu et al. trace the skeletal stem/progenitor transition during postnatal bone formation using a dual-recombinase lineage-tracing systems and show that osteoblasts and BMSCs in the marrow cavity may arise from the growth plate before adolescence [28]. Given that GP-SSCs contribute to osteoblast formation, bone volume changes may be associated with aberrant differentiation of early GP-SSCs and not just aberrant osteogenic differentiation of BMSCs, though it is unclear whether GP-SSCs have a major or more limited contribution to the osteoblast pool.

## Periosteum-derived skeletal stem cells (P-SSCs)

The periosteum is the connective tissue covering the outer surface of bone and has a complex cellular composition including osteoprogenitor cells located in the inner layer, immune cells, fibroblasts, blood vessels, and nerves [46]. The periosteum is essential for cortical bone maintenance and repair, while the growth plate plays a major role in bone elongation. However, given that many Cre lines label a broad population of stromal cells, it has not been determined which population within the periosteum has represents periosteal SSCs. P-SSCs undergo intramembranous ossification to produce osteoblasts under physiologic conditions, however fracture healing induces plasticity in this population whereby they acquire both the markers and endochondral ossification capacity of endosteal osteoblasts [6, 9, 28, 47, 48]. Surprisingly, Cathepsin K (Ctsk), which was earlier used as a marker for bone-resorbing osteoclasts, is found to label periosteal stem cells mediating intramembranous ossification when used in combination with other cell surface markers [6, 49]. Specifically, CD45^−^TER119^−^CD31^−^CD105^−^CD200^+^ cell subsets isolated from the Ctsk-Cre-labeled cell populations in the femoral periosteum of young mice exhibit self-renewal during serial *in vivo* transplantation, and this specific cell type sits at the apex of their differentiation hierarchy, being able to generate all of the Ctsk-Cre labeled skeletal cell types. The finding that these P-SSCs also express the Chan et al. markers raises the possibility that this surface immunophenotype may be shared by multiple types of SSCs or even represent a general signature of SSCs. *In vivo* deletion of the osteogenic differentiation transcription factor Osx in Ctsk-Cre-labeled cells resulted in poor fracture repair and impaired formation of the bone cortex. Furthermore, human periosteal cells from femur express Ctsk and CD200 by immunostaining. Lin^−^CD90^−^CD200^+^CD105^−^ SSCs in human periosteum are multipotent *in vitro* and mediate intramembranous bone formation by renal capsule transplantation experiment of immunocompromised mice [6].

## Other potential bone forming stem/progenitor cells

Interestingly, heterotopic ossification (HO) results in the formation of bone-like structure containing bone and cartilage in soft tissues like muscle and tendon, which prompts that bone-forming stem/progenitor cells exist in these tissues [50]. PDGFRα-Cre, Tie2-Cre, and Mx1-Cre labeled fibro/adipogenic progenitors (FAPs) drive the HO formation via endochondral ossification under the specific condition of abnormal activation of signaling pathway [51–53]. In parallel, FAPs are a major source of adipocytes in muscle. Surprisingly, FAPs in muscle supply osteoblasts and chondrocytes in the callus during fracture healing [54]. Scleraxis (Scx) is the key transcriptional factor for tendon development and Scx-Cre/CreER can label tendon progenitor cells (TPCs). Scx^+^ TPCs have the clonal forming ability and multi-lineage differentiation potential contributing to fibroblasts, osteoblasts, and chondrocytes [55, 56]. However, it remains unclear which cells in the Scx-lineage represent tendon/ligament stem cells. Though these bone-forming stem/progenitor cells belonging to motor system are not located in the bone, further study of these cells may help to provide a novel cell source for SSC therapy.

## The SSC niche

R. Schofield defined “stem cell niche” as a specific microenvironment that promotes stem cells renewal and maintains the stemness properties in 1978 [57]. These microenvironmental components of stem cell niches are variable, including interactions with neighboring cell types, intricate networks and gradients of signaling molecules, extracellular matrix components, and various biochemical and biophysical cues [58, 59]. Stem cells are the core component of tissue regeneration, and studying their stem cell microenvironment is of great importance for the study of regenerative medicine. Below, we summarize and discuss the reported stem cell microenvironments of BMSCs and SSCs.

## The bone marrow niche of BMSCs

**Figure 2.**
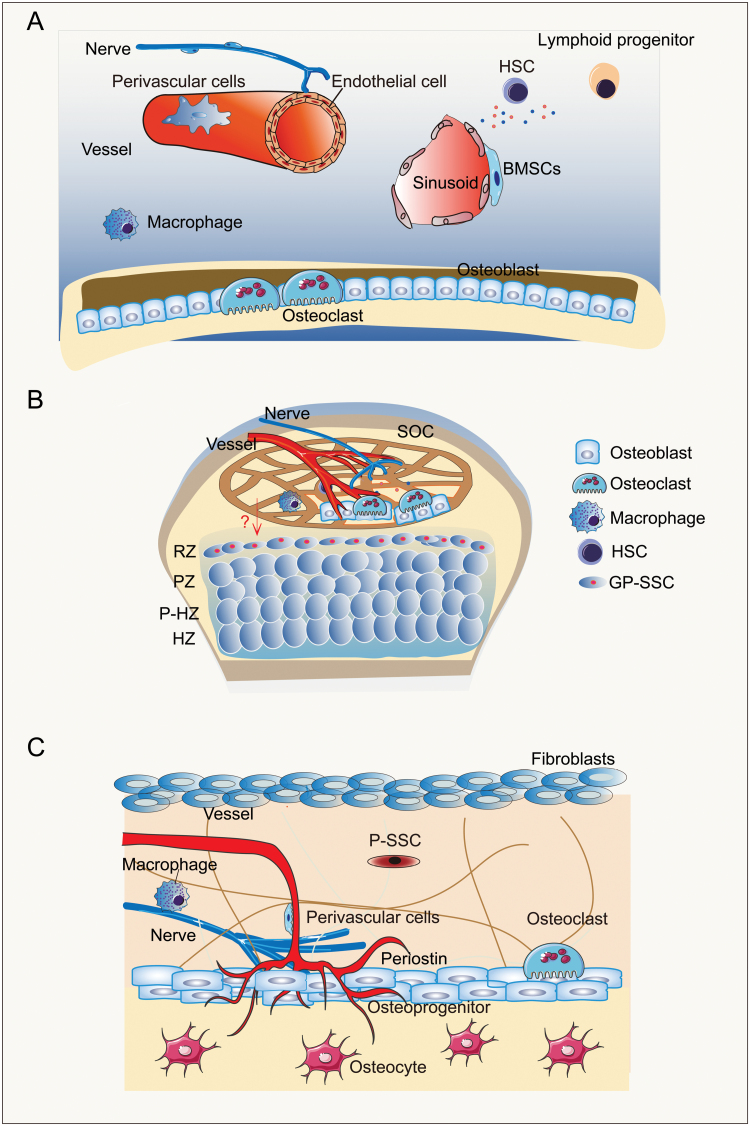
The potential niches of SSCs. (A) Cell types regulating BMSC niches include sympathetic nerve cells, macrophages, and osteoclasts, osteoblasts, CXCL12-abundant reticular (CAR) cells, hematopoietic stem cells, lymphoid progenitors and endothelial cells, etc. (B) The niches of BMSCs are formed by inmune cells, osteo-lineage cells, and other components (nerves and vessels) of the secondary ossification center adjacent to the resting zone in the growth plate. (C) The periosteum contains small blood vessels that provide nourishment to the outer cortex of bones and has a rich supply of sensory nerves to perceive pain. Extracellular matrix (periostin), osteoblasts, osteocytes, fibroblasts, immune cells, blood vessels, and sensory nerves probably participate in the formation of P-SSC niches.

The bone marrow microenvironment is a very complex system including lymphoid cells, endothelial cells, HSCs, macrophages, adipocytes, osteoblasts, osteoclasts, osteocytes, CAR cells, etc [17, 59] (Fig. 2A). Early studies of HSC niche found that LepR labels perivascular BMSCs. LepR^+^ BMSCs can regulate the maintenance of HSCs by secreting Scf and Cxcl12, suggesting that BMSCs themselves can secrete cytokines to influence the HSC niche [36, 40]. Peri-arteriolar LepR^+^Oln^+^ cells are a subpopulation of LepR^+^ BMSCs supporting osteogenesis. Interestingly, conditional ablation of Scf in adult Oln^+^ cells impaired lymphopoiesis instead of the maintenance of HSCs. Deletion of mechanosensor channel PIEZO1 in Oln^+^ cells diminished Oln^+^ cells and common lymphoid progenitors (CLPs), indicating the importance of mechanical niche for osteogenesis and lymphopoiesis in bone marrow [20].

Blood vessels in the bone marrow are critical in regulating BMSC fate. In bone marrow, LepR^+^ and Cxcl12^+^ BMSCs are located around the arterioles, suggesting a possible direct interaction between them and blood vessels [19, 22]. After reducing the number of type H vessels (CD31^hi^EMCN^hi^) in the bone by knocking down HIF-1α in endothelial cells, the number of osteoprogenitor cells also decreased, resulting in damage to the trabecular bone structure and thinning of the bone cortex [60]. In addition, type H blood vessels are also regulated by Slit homologous protein 3 (SLIT3), suggesting a possible role of SLIT3 in regulating the BMSC niche to influence osteogenesis [61].

HSCs typically reside in the bone marrow niche of adult skeleton, and the aging of HSCs and the hematopoietic system may be associated with skeletal aging. Aging BMSCs in mice alters components and signaling in the bone marrow niche and distorts differentiation of BMSCs and HSCs, resulting in poor regeneration of bones [62]. With decreased differentiation potential into osteoblasts and chondrocytes, aging BMSCs secrete plenty of pro-inflammatory and pro-resorptive cytokines like CSF1 and CCL11. Senescent BMSC lineages promote osteoclast activity and increase the myelopoietic versus lymphopoietic output of hematopoietic stem/progenitor cells, demonstrating that BMSC aging is in turn a driver the overall aging of the HSCs niche [62].

Bone tissue comprises sympathetic and sensory nerves, and both of these neuro-secreted factors have been found to regulate osteogenesis, suggesting that nerves may regulate the fate of BMSCs in the bone marrow [63–65]. Leptin was reported to have an influence on bone mass by regulating sympathetic nerves. This led to the secretion of norepinephrine (NE) from peripheral nerves to the local microenvironment, which in turn activated β-adrenoceptors expressed by osteoblasts [66]. Moreover, conditional deletion of LepR in Prrx1-Cre-labeled BMSCs leads to increased bone formation and decreased adipogenesis, showing that Leptin/LepR signaling regulates adipogenesis and osteogenesis of BMSCs in the bone marrow [67]. Calcitonin gene‐related peptide (CGRP) is the neuropeptide primarily secreted by sensory nerves that regulates osteoblast differentiation. Receptors for CGRP are enriched in BMSCs during ostegenesis, and CGRP promote osteogenic differentiation of BMSCs by activating the Wnt/β‐catenin signaling pathway [68]. CGRP concentration increases after bone compression, suggesting a role for sensory nerves in the regulation of bone responsing to mechanical stress [69]. Substance P (SP) secreted by sensory nerves, can promote osteoblast differentiation by increasing cAMP production and enhancing BMP2 secretion [70, 71]. Neuropeptide Y (NPY) is a vasoconstricting peptide that often work in combination with NE in sympathetic nerves, which has been reported to affect bone metabolism [72]. These studies demonstrate the distinct regulatory roles of neuronal factors on BMSCs, but mechanisms involved are still need further *in vivo* study [73, 74].

Immune cells have been demonstrated to play a key role in regulating the microenvironment of bone marrow. Intramedullary macrophages were found to regulate bone and adipogenesis by secreting a pro-osteogenic factor, Reticulocalbin-2 (RCN2), in response to mechanical force [75]. Furthermore, macrophages and neutrophils increase and secrete abundant grancalcin in the bone marrow during aging in mice and rats. Grancalcin was capable of inducing premature skeletal aging of young mice and conditional deletion of it in macrophages and neutrophils resisted skeletal aging [76]. These suggest that macrophage-derived factors regulate the BMSC microenvironment and are essential for osteogenesis.

## The growth plate SSC niche

The growth plate stem cell niche, also known as epiphyseal stem cell niche, is formed in part by the SOC due to the close spatial proximity of GP-SSCs and SOCs [31, 77]. It is likely that the many local cell types or acellular niche components including extracellular matrix, osteoblasts, osteocytes, mesenchymal cells, hematopoietic lineage cells, and endothelial cells all participate in regulating the GP-SSCs niche. Furthermore, mechanical loading, systemically secreted factors also likely act to maintain the GP-SSC niche (Fig. 2B).

Hedgehog (HH) signaling is critical in maintaining growth plate development and homeostasis [78]. SHH and IHH are two important diffusible morphogens that activate HH signaling [78, 79]. By analysis of SHH-GFP transgenic mice, mesenchymal stem/progenitor cells, endothelial cells, and hematopoietic cells express abundant SHH in the SOC adjacent to GP-SSCs.

Given that Pthrp is a marker of GP-SSCs, and Pthrp expression is classically regulated by IHH in a counter-gradient, the diffusion of IHH from the prehypertrophic zone is likely to regulate the maintenance of GP-SSCs [31, 77, 80–85]. Indeed, treatment with hedgehog inhibitors results in premature growth plate fusion, suggesting that this fusion process may be the direct result of depleting the GP-SSCs [86–89]. Overexpression of SHH in Col2a1-Cre-labeled chondrocytes promotes chondrocyte proliferation, inhibits chondrocyte hypertrophy, and suppresses the formation of trabecular bone formation [90]. Postnatal inactivation of IHH led to the disorganized columnar structure and enhanced hypertrophic differentiation of chondrocytes in the growth plate [81, 91–93]. In conclusion, HH signaling is clearly involved in the regulation of stem cell plasticity, but the direct and detailed role of HH ligands in the niche and in GP-SSCs remains to be elucidated.

Pre-hypertrophic chondrocytes, the progeny of GP-SSCs are a major source of IHH [81, 91]. Therefore, the progeny of GP-SSCs are involved in stem cell niche formation in a paracrine manner. The IHH-PTHrP axis is critical for growth plate maintenance, with PTHrP delaying hypertrophic differentiation of proliferating chondrocytes and IHH promoting chondrocyte proliferation [41, 82, 85]. Gradients of distinct morphogens appear to play important roles in the GP-SSC niche, but studies on components of the SOC regulating stem cell fate are still lacking.

## The periosteal SSC niche

The periosteum refers to a fibrous connective tissue membrane that covers the surfaces of all bones except cartilage surfaces. It consists of two layers: an outer fibrous layer and an inner cellular layer. The periosteum contains small blood vessels that provide nourishment to the outer cortex of bones and has a rich supply of sensory nerves to perceive pain. The inner layer of periosteum contains P-SSCs which are involved in bone development, continuous remodeling, and repair. Extracellular matrix, osteoblasts, osteocytes, fibroblasts, immune cells, blood vessels, and sensory nerves probably participate in the formation of P-SSC niches [48, 94] (Fig. 2C).

Once a fracture occurs, the nerve is initially recruited to the site of injury. Nerves that release vasoconstrictor and osteogenic factors are indispensable for bone fracture healing. It has been reported that nerves play a key role in regulating the plasticity of P-SSCs. There are clinical data showing that concomitant traumatic brain injury (TBI) promotes bone healing [95]. One mechanism of repair is that injured neurons release miRNA-enriched extracellular vesicles targeting osteoprogenitors in bone to stimulate bone formation [96]. The tropomyosin receptor kinase A-expressing (TrkA-expressing) sensory neurons transmits nociceptive signals by nerve growth factor (NGF) to mediate bone pain. NGF is significantly upregulated acutely after bone injury, and the disruption of NGF/TrkA signaling inhibits bone repair [97–99]. Mice lacking NGF in myeloid cells (LysM-Cre; NGF^flox/flox^ mice) showed impaired cranial bone repair compared with control mice. NGF within a nascent callus may derive from PDGFRα/β^+^ cells and F4/80^+^ macrophages by immunofluorescence staining, which may provide the evidence that P-SSCs modulate periosteal stem cell niche through autocrine and paracrine manner [100].

Blood vessels surrounding periosteum supply oxygen and nutrient for P-SSCs. In the early stages of bone injury, vascular endothelial cells begin to expand and form blood vessels within the callus [100]. Around 10% of patients with bone fractures show impaired fracture healing for lack of blood supply, revealing that blood vessels is essential to bone fracture healing [101, 102]. Pharmacological inhibition of angiogenesis leads to fibrosis of callus and prevents cartilaginous callus formation in fracture healing [103]. P-SSCs undergo chondrogenic differentiation instead of osteogenic differentiation when blood supply is impaired, which might prompt that nutrients supplied by the blood vessels regulate cell fate of P-SSCs [104–106]. It has been shown that lipids from local vasculature influences the differentiation of P-SSCs during fracture healing. P-SSCs upregulate the expression of Sox9 by promoting forkhead box O (FOXO) transcription binding to the Sox9 promoter when lipids are scarce, which induces chondrogenic commitment [107]. Thus, the metabolic profile of P-SSCs deserves to be studied, which may reflect microenvironmental constraints as well as specific cellular requirements and help to identify important regulators of stem cell fate. Furthermore, numerous factors and cytokines contribute to bone vascularization including VEGF, placental growth factor (PIGF), FGF, PDGF and insulin-like growth factor (IGF) [105, 108–111]. Osteochondral progenitor cells and hypertrophic chondrocytes-derived VEGFs recruit blood vessels and osteoclasts during the periosteal endochondral ossification stage [112]. However, the cell sources of those factors and cytokines contributing to bone vascularization remain elusive.

ECM molecules are crucial for regulating P-SSC function and bone repair. Periostin (Postn), an ECM protein in the inner layer of the periosteum regulating cell–cell and cell–matrix interactions is indispensable to bone repair. In response to bone injury, Postn and other Postn-related ECM proteins are upregulated in Prrx1-Cre-labeled periosteal cells. Bone regeneration and callus size and quality in mice lacking Postn are all reduced. Interestingly, the transplantation of Postn-knockout (Postn-KO) periosteal cells in Postn-KO mice made bone repair more difficult after a second injury, further supporting that Postn and Postn-related ECM proteins may be involved in activation of P-SSCs and stem cell niche regulation in response to injury. The deletion of Postn affects the expression other ECM molecules in Postn-KO Prrx1-Cre-labeled periosteal cells, suggesting complementary roles of these ECM molecules in activation of SSCs [9].

Macrophages are essential for bone repair because bones do not heal effectively when depleted. Macrophages are recruited to the injured sites after trauma and then function with phenotypic changes. Macrophages promote bone fracture healing by secreting vasoregulatory, neurotrophic, and osteochondral factors like VEGF, NGF, TGFβ1, etc [100, 113–116]. Combined with bone marrow transplantation experiment, heterochronic parabiosis experiment suggests that macrophages from young mice rejuvenate fracture repair, and macrophages from old mice delay bone healing in young mice. Low density lipoprotein receptor-related protein 1 (Lrp1) produced by young macrophages is identified to promote osteoblast differentiation through the proteomic analysis of the secretomes [117]. In addition, macrophage-lineage tartrate-resistant acid phosphatase-positive cells stimulate the expression of Postn and recruit of P-SSCs to the periosteal surface through the secretion of platelet-derived growth factor-BB (PDGF-BB). Moreover, deletion of PDGFRβ (the receptor for PDGF-B) in the LepR-lineage cells impairs periosteal bone formation and regeneration [118].

Ccl5- and Ccr5-defificient mice display significant reduction of new bone formation and external callus volume at defect sites after injury, showing the importance of chemokine for bone repair [29]. Besides chemokines, secreted factor Bmp2 has been reported to be critical for fracture repair. Bmp2-deficiency in Prrx1-lineage cells leads to spontaneous fractures in mice [119]. Interestingly, the study also found that Osx-Cre; Bmp2^flox/flox^ mice had no fracture repair phenotype, which provides the evidence that Bmp2 from Prrx1^+^Osx^−^ cells is key to bone fracture healing [120]. IHH is enriched in the cells of callus and may also contribute to the healing of fractures by periosteal cells [121]. *In vitro* activation of HH signaling promotes the osteogenic and chondrogenic differentiation of periosteal stem/progenitor cells. Inactivation of HH signaling by deleting Smoothened (frizzled class receptor gene Smo) attenuates cartilaginous callus formation after fracture and diminishes callus cell proliferation [122, 123]. Retinoic acid (RA) signaling is critical for skeletal development and postnatal maintenance of the skeletal system [124]. Clinical data indicate that serum retinol levels are associated with the probability of fracture [125]. Enhanced RA regulated by histone demethylase LSD1 could decrease SOX9 levels of Prrx1-lineage cells, indicating RA signaling may have a negative effect on chondrocyte differentiation of P-SSCs [27]. Mechanical loading is critical for P-SSC niche as it significantly promotes cortical bone formation by increasing the number of myeloid‐lineage cells and the expression of active transforming growth factor β (TGF‐β). Mechanical loading-induced periosteal bone formation can be inhibited by knockout of Tgfb1 in myeloid‐lineage cells in mice [114]. Myeloid‐lineage cells are one of the important components of periosteal mechanical microenvironment of the periosteum and can sense mechanical stimuli to secrete TGF-β1 for osteogenesis. Nonetheless, the mechanisms of the mechanosensing interaction between P-SSC and niche remain to be clarified.

## Signaling pathways in SSCs during bone development and repair

Bone development and injury repair are regulated by multiple signaling pathways, and the complex signaling is also present in the regulation of plasticity of SSCs. Despite the entirely different upstream signals or downstream targets, the different signaling pathways act in a coordinated manner to ensure the proper function of SSCs in the bone development and regeneration.

## HH signaling

**Figure 3.**
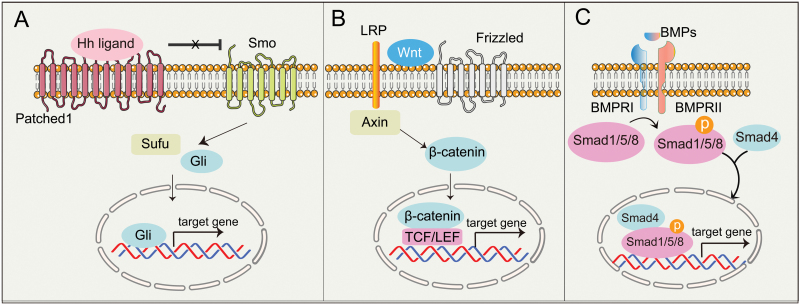
The regulatory signaling of SSCs during bone development and repair. (A) HH ligands bind to Patched protein (PTCH1) and remove its repression of SMO. SMO is free to promote the movement into the cell nucleus of GLI protein, which are converted from their repressed to activated forms and drive osteochondral gene expression. (B) The secreted WNT ligands bind to the membrane surface receptor protein Frizzled (FZD) and activate the intracellular protein DVL. DVL stabilizes the free state of β-catenin protein in the cytoplasm and promotes stably accumulated β-catenin in the cytoplasm enters the nucleus and binds to the LEF/TCF transcription factor family to initiate transcription of downstream target genes. (C) BMPs bind to BMP receptors (BMPRI/BMPRII), leading to phosphorylation of SMAD proteins (SMAD1, SMAD5, or SMAD8). The SMAD proteins form a complex and bind to SMAD4, which then enters the nucleus to regulate gene expression.

Canonical HH signaling pathway involves the expression of three HH ligands, namely Sonic hedgehog (SHH), Indian hedgehog (IHH), and Desert hedgehog (DHH). All HH ligands bind to Patched protein (PTCH1), which can inhibit Smoothened (SMO). After binding with Hh ligands, Ptch1 derepresses Smo, allowing the full-length Gli protein to enter the nucleus to activate transcription of downstream target genes [79] (Fig. 3A).

HH signaling has long been shown as a key regulator in bone development and repair [126]. IHH maintain the appropriate differentiation of chondrocytes by regulating Parathyroid hormone-related protein (PTHrP) and other ways [83]. The fate of Pthrp^+^ GP-SSCs is regulated by IHH and PTHrP signaling [26]. Deletion of the tyrosine phosphatase SHP2 (encoded by PTPN11) in Col2a1-CreER-labeled cells induces chondrocyte proliferation in the growth plate. IHH levels increased significantly in SHP2-depleted cells *in vitro and vivo*, and an inhibitor of IHH signaling reduced the chondrocyte proliferation [127, 128]. A microarray analysis reported that the expression of the protein regulating IHH secretion named Dispatched-1 (Disp1) and IHH, increased in BCSPs of callus in 7 dpf (days post fracture), whereas HH interacting protein Hhip, an IHH pathway antagonist, is down-regulated [129]. The same group also discovered that high serum concentrations of TNFα in diabetes mellitus mice could repress the expression of IHH in SSCs, resulting in inhibited SSCs expansion and impaired bone fracture healing. In addition, by delivery of exogenous IHH in the fracture site, the expansion and differentiation potential of SSCs and bone healing were restored [130]. SLIT and NTRK-like protein-5 (Slitrk5), a transmembrane protein is a negative regulator of HH signaling. Loss of SLITRK5 enhances HH signaling and osteoblast differentiation to improve bone fracture healing [131].

## WNT signaling

The WNT signaling pathway plays a key role to ensure normal bone development and homeostasis. The secreted WNT ligands bind to the membrane surface receptor protein Frizzled (FZD) and activate the intracellular protein DVL. DVL stabilizes the free state of β-catenin protein in the cytoplasm by inhibiting the degradation activity of the β-catenin degradation complex formed by GSK3β and other proteins. Stably accumulated β-catenin in the cytoplasm enters the nucleus and binds to the LEF/TCF transcription factor family to initiate transcription of downstream target genes [42, 132] (Fig. 3B). Conditional deletion of β-catenin in Cxcl12-CreER-labeled BMSCs impaired cortical bone regeneration [22]. Beside its functions in BMSCs, WNT signaling also plays a vital role in regulating the function of SSCs. Activation of WNT signaling in Pthrp^+^ GP-SSCs impairs the formation of columnar chondrocytes in the growth plate by analyzing the phenotype of Pthrp-CreER; Apc^flox/flox^ mice [133]. Gene-expression profiling revealed that CD45^−^/TER119^−^/Tie2^−^/Thy^−^/6C3^−^/CD105^−^/AlphaV^+^/CD200^+^ GP-SSCs express both receptors involved in WNT signaling pathway, and diffusible ligands of the pathway, including Wnt3a [5]. Interestingly, Ctsk^+^ P-SSCs are enriched in the expression of WNT ligands (Wnt4, Wnt11, Wnt5a, Wnt5b) compared with non-Ctsk MSC [6]. Therefore, these may infer a potential role for WNT signaling pathway in regulating SSCs.

## BMP signaling

Bone morphogenetic proteins (BMPs) belong to the transforming growth factor-β (TGF-β) superfamily, which also comprises TGF-βs subfamily, the activin subfamily, and other related proteins. TGF-βs and BMPs act on serine/threonine-specific protein kinase receptors on the cell surface to signal to both classical Smad-dependent and non-classical Smad-independent pathways (p38 mitogen-activated protein kinase/p38 MAPK) to regulate skeletal development and homeostasis [134] (Fig. 3C).

In view of the fundamental role of BMP signaling in osteogenic differentiation both in bone development and repair, BMP signaling might have an important effect on the SSC function [119, 135]. Postnatal the maintenance of normal bone width in mice requires BMP signaling at the periosteal niche. Interestingly, mice with conditional deletion of Bmp2 in Prrx1^+^ skeletal stem/progenitors (Prrx1-Cre; Bmp2^flox/flox^), but not Osx^+^ osteoprogenitors or Col1a1^+^ mature osteoblasts showed periosteal growth defects [136]. Indeed, single-cell sequencing of mouse SSCs revealed the co-expression of BMP2 and its receptor, suggesting the potential of autocrine and/or paracrine BMP signaling in the SSCs. BMP2 could rapidly induced expansion of SSCs *in vitro*, but TGF-β did not. mSSC can differentiate to form ectopic bone with marrow induced by BMP2 in the subcutaneous fat pads. Interestingly, the progeny of SSCs exhibited decreased expression of BMP2 but increased expression of BMP2 antagonists, including Noggin and Gremlin 2, suggesting a negative feedback loop of the BMP2 signaling in the regulation of SSCs [5]. In addition, BMP2 and Smad3 are also upregulated in SSCs during bone fracture, which is essential for bone healing [137]. Recent studies also demonstrated the great potential of delivering exogenous BMPs in injury sites to enhance the function of endogenous SSCs and promote bone healing [138, 139].

## SSCs-related clinical therapy

The number of SSCs declines with age, accompanied by gradually reduced bone formation capacity [140]. The dysfunction of SSCs can also lead to skeletal diseases like delayed fracture healing or nonunion, bone tumors, etc. Deletion of Osx in Ctsk^+^ P-SSCs of Ctsk-Cre; Osx^flox/flox^ mice lead to markedly impaired fracture healing with a significant decrease of callus formation showing that SSCs are indispensable for bone repair [6]. Benign bone tumors contain two main categories: bone-forming lesions (e.g., osteoid osteoma, osteoblastoma) and cartilage-forming lesions (e.g., osteochondroma, enchondroma) [141]. The cell origin of bone tumors remains elusive. However, evidence suggests that SSCs or their progeny may be an important source of bone tumors. Deletion of tumor suppressor gene Liver kinase b1 (LKB1) in Ctsk^+^ P-SSCs causes the excessive proliferation and abnormal differentiation Ctsk^+^ P-SSCs by activating mTORC1 signaling, which leads to osteogenic tumors upon cortical bone. Knockout Raptor (a core binding factor of mTORC1) or drug delivery of an mTORC1 inhibitor Rapamycin, can ameliorate the progression of ostegenic tumor [142]. In addition, deleting SHP2 (encoded by PTPN11) in Ctsk^+^ stem/progenitors in perichondrium causes cartilage-forming lesions by activating HH signaling. The suppression of HH signaling ameliorates the progression of cartilage-forming lesions. These studies show that targeting SSCs or their progeny may be a promising and effective therapy.

Interestingly, activation and inactivation of the same signaling pathway can cause phenotypically opposite skeletal diseases, for example, activation of BMP signaling in stem/progenitor cells can lead to HO in soft tissue, yet inactivation of BMP signaling can cause nonunion [51, 119, 143]. Activation of HH signaling in stem/progenitor cells leads to chondroma and HO, whereas inactivation of HH signaling causes poor fracture healing and skeletal shortening [81, 85, 89, 123, 127, 144, 145]. Activation of mTOR signaling in SSCs causes osteogenic tumors, and rapamycin, an inhibitor of mTOR signaling, can effectively alleviate this phenotype. In addition, rapamycin can also be used as a potential drug for the treatment of HO [142, 146, 147]. Thus, the comparative mechanisms of different diseases may shed light on the treatment of skeletal diseases potential strategies for the treatment of skeletal diseases.

Stem cells hold great promise for clinical applications. Various types of MSCs have been identified and manipulated including BMSCs and other cell population like adipose-derived MSCs (ASCs), and genetically modified MSCs [148]. Currently, SSC and BMSC-based therapies are at an early stage of development given the recent discovery of these populations, though they do hold promise in conditions where it is paucity of these cellular populations themselves or a cell intrinsic functional defect that underlies disease. Future studies will need to be conducted with well-defined cellular therapy products and focus on mechanistic endpoints that most notably include assessment of cellular engraftment.

Combined growth factors with stem cell-based therapy offers a promising approach for the development of complex biological therapies. BMP2 protein has been an effective FDA-approved drug for the treatment of poor clinical fracture healing, suggesting that inhibitors and agonists of different pathways are expected to be potential drugs for SSCs dysfunctional diseases. Following micro-injury of joint, SSCs are activated to form fibrocartilage, but co-delivery of BMP2 and VEGFR inhibitor reverses this process and promotes differentiation towards cartilage [149]. SSCs can be induced to generate cartilage for treatment of localized chondral disease in osteoarthritis with defined factors. In parallel, stem cells of other sources, derivatives of stem cells like growth factors, and cellular modification methods should be explored to determine the optimum conditions for cell therapy.

## Conclusions and perspectives

This review systematically summarizes the different regions of SSCs and identifies the contribution of different cell types in skeletal development and repair. All three types of cells (P-SSCs, GP-SSCs, and BMSCs) are capable of long-term persistence. In light of previous studies, the origin of the three sources of stem cells is yet to be clarified. Barcode-based single-cell lineage tracing may serve as a good technique to solve this problem. Single-cell lineage tracing can determine the relationship between ancestral and progeny cells using barcode evolution and define cell identity combined with single-cell RNA sequencing, ultimately dissecting cell origin and reflecting hierarchical relationships [150]. The technology is now well established for the study of HSC lineage and has updated previous knowledge on the origin of HSCs [151, 152].

With innovations in single-cell sequencing and transgenic animal technology, more and more subpopulations of SSCs have been identified, but many important issues remain in this field: (1) the identification of the specific SSC markers. It is found that the previously identified SSCs are heterogeneous by single-cell sequencing, therefore the inclusion relationships of known SSC subpopulation remains to be clarified. More specific cell surface makers and the location of SSCs remain to be identified; (2) the regulatory mechanisms of SSC fate still need to be explored, for example, signaling pathways regulate the differentiation of SSCs in the resting zone into hypertrophic chondrocytes, osteoblasts, and BMSCs remain unclear. And regulatory mechanisms of microenvironmental components such as immune cells, endothelial cells, and fibroblasts involved in cell fate of SSCs, remain to be elucidated. Dissecting the dynamics of microenvironment components of SSCs and mapping regulatory networks of “SSCs-niche” and “niche-niche” under the different conditions (stress, aging, diseases) the focus of later studies; (3) the scientific and technical hurdles lead to inadequate stem cell sources for transplantations. Developing small molecules/proteins targeting endogenous SSCs to promote their proliferation and differentiation could be an alternative therapy for certain type of SSCs-related skeletal system disorders. (4) There is still a lack of single-cell sequencing profiles of human skeletal development, injury and aging processes, as well as models for preclinical studies. Overall, a better understanding of the plasticity and niches of SSCs surely will provide novel therapeutic approaches clinically.
